# Cerebral blood flow response to acute hypoxic hypoxia

**DOI:** 10.1002/nbm.3026

**Published:** 2013-10-07

**Authors:** Ashley D Harris, Kevin Murphy, Claris M Diaz, Neeraj Saxena, Judith E Hall, Thomas T Liu, Richard G Wise

**Affiliations:** a CUBRIC, School of Psychology, Cardiff UniversityCardiff, UK; b Department of Anaesthetics, Intensive Care and Pain Medicine, School of Medicine, Cardiff UniversityCardiff, UK; c Center for Functional Magnetic Resonance Imaging and Department of Radiology, University of California San DiegoLa Jolla, CA, USA

**Keywords:** arterial spin labelling (ASL), blood oxygenation, cerebral blood flow (CBF), cerebral perfusion, hypoxia, *R*_2_*, temporal dynamics

## Abstract

Hypoxic hypoxia (inspiratory hypoxia) stimulates an increase in cerebral blood flow (CBF) maintaining oxygen delivery to the brain. However, this response, particularly at the tissue level, is not well characterised. This study quantifies the CBF response to acute hypoxic hypoxia in healthy subjects. A 20-min hypoxic (mean *P*_ET_o_2_ = 52 mmHg) challenge was induced and controlled by dynamic end-tidal forcing whilst CBF was measured using pulsed arterial spin labelling perfusion MRI. The rate constant, temporal delay and magnitude of the CBF response were characterised using an exponential model for whole-brain and regional grey matter. Grey matter CBF increased from 76.1 mL/100 g/min (95% confidence interval (CI) of fitting: 75.5 mL/100 g/min, 76.7 mL/100 g/min) to 87.8 mL/100 g/min (95% CI: 86.7 mL/100 g/min, 89.6 mL/100 g/min) during hypoxia, and the temporal delay and rate constant for the response to hypoxia were 185 s (95% CI: 132 s, 230 s) and 0.0035 s^–1^ (95% CI: 0.0019 s^–1^, 0.0046 s^–1^), respectively. Recovery from hypoxia was faster with a delay of 20 s (95% CI: –38 s, 38 s) and a rate constant of 0.0069 s^–1^ (95% CI: 0.0020 s^–1^, 0.0103 s^–1^). *R*_2_*, an index of blood oxygenation obtained simultaneously with the CBF measurement, increased from 30.33 s^–1^ (CI: 30.31 s^–1^, 30.34 s^–1^) to 31.48 s^–1^ (CI: 31.47 s^–1^, 31.49 s^–1^) with hypoxia. The delay and rate constant for changes in *R*_2_* were 24 s (95% CI: 21 s, 26 s) and 0.0392 s^–1^ (95% CI: 0.0333 s^–1^, 0.045 s^–1^ ), respectively, for the hypoxic response, and 12 s (95% CI: 10 s, 13 s) and 0.0921 s^–1^ (95% CI: 0.0744 s^–1^, 0.1098 s^–1^/) during the return to normoxia, confirming rapid changes in blood oxygenation with the end-tidal forcing system. CBF and *R*_2_* reactivity to hypoxia differed between subjects, but only *R*_2_* reactivity to hypoxia differed significantly between brain regions. © 2013 The Authors. *NMR in Biomedicine* published by John Wiley & Sons, Ltd.

## INTRODUCTION

An acute decrease in the arterial partial pressure of oxygen (*P*_a_o_2_) stimulates increased blood flow to the brain to maintain cerebral oxygen delivery. However, the regional perfusion increase can be subtle during mild hypoxia [arterial *P*o_2_ range of 60–150 mmHg 1], only becoming more pronounced and consistent with moderate to severe hypoxic challenges 2. The blood flow response to hypoxia is also dynamic as it evolves with prolonged hypoxic exposure, for example, over the course of days 1,3.

One of the first methods to measure blood flow to the brain was the Kety–Schmidt method, which is based on indicator diffusion theory 4. It was used to investigate the effects of altered blood gases on blood flow to the brain 5 and found that blood flow increased by 35% with a 10% inspired oxygen challenge. Currently, one of the most widely used techniques for the assessment of alterations in bulk blood flow is transcranial Doppler (TCD) ultrasound. TCD measures velocity directly and relies on the relationship between the arterial blood velocity and blood flow, and typically assumes a constant vessel cross-sectional area. The blood flow response to a range of levels of acute and chronic hypoxia, as well as the sensitivity of blood flow to hypoxic hypoxia at different levels of end-tidal CO_2_, have been characterized using TCD ultrasound 1,3,6. Reactivity to hypoxia and hypercapnia of different arteries feeding the brain, specifically the internal carotids, the vertebral arteries and the middle (MCA) and posterior cerebral arteries, has been characterised using TCD 7. The temporal dynamics of bulk blood flow in the MCA during acute hypoxic hypoxia have been characterised with TCD, suggesting that the time constant in response to hypoxia is approximately 80 s and the time constant for return to normoxia after hypoxia is 29 s with a delay in the initiation of each response of 5 s 8.

In contrast with the measurement of bulk arterial flow, cerebral tissue perfusion can be measured using nuclear imaging methods (positron emission tomography or single photon emission computed tomography), computed tomography as well as MR-based methods. With a 12% inspired oxygen challenge, global cerebral blood flow (CBF) has been shown to increase by 8.7% 9. CBF in some brain regions has been shown to be more responsive to hypoxia than in others 10,11. Binks *et al*. 11 provided evidence that phylogenetically older regions of the brain, such as the basal ganglia, putamen, caudate and pallidum, have a relatively larger CBF increase in response to hypoxia than ‘newer’ brain regions. Pagani *et al*. 10 found a different set of regions with comparatively greater CBF in response to hypoxia, specifically the anterior cingulate cortex, right temporal lobe, sensory motor cortices, prefrontal cortex and the basal ganglia. Regional differences in the sensitivity of the CBF response to hypoxia may have implications for regional susceptibility to damage or adaptation to hypoxic conditions. As an alternative imaging technique, arterial spin labelling (ASL) MRI provides a method to investigate CBF that does not require the injection of an exogenous intravascular contrast agent. In addition, ASL can be performed continuously and is therefore suitable for the characterisation of the temporal parameters of the CBF response to hypoxia.

A couple of ASL-based MRI studies have estimated the magnitude of the CBF response to hypoxic hypoxia. In one study 12, the hypoxic challenge was defined by a 9–14% decrease in blood oxygen saturation, with imaging performed after the end-tidal oxygen levels had stabilized for several minutes; however, variable CBF responses were observed. Although there was a statistically significant increase in CBF (7% increase in CBF per 10% drop in arterial oxygen saturation), 30% of the subjects showed a negative response, specifically a drop in CBF with hypoxia. In an examination of the CBF response to investigate acute mountain sickness susceptibility 13, CBF was shown to increase by 11–16% in grey matter during a 30-min fixed 12.5% inspired O_2_ challenge. Although, in this study, an explicit description of the temporal CBF response to acute hypoxia was not given, it appears that the increase in CBF took longer than the relatively rapid response (less than 2 min) described by Poulin *et al*. 8 using TCD.

The objective of this study was to quantify the magnitude and temporal dynamics of the CBF response to hypoxic hypoxia for whole-brain grey matter and on a regional basis. *R*_2_*, being sensitive to blood deoxyhaemoglobin content and therefore a marker of local blood oxygenation 14, was quantified to examine the relationship between altered blood oxygenation and CBF. We measured CBF (using ASL) continuously during a 33-min protocol that included rest, a 20-min inspiratory hypoxic challenge and a normoxic recovery phase in healthy volunteers.

## MATERIALS AND METHODS

Twelve healthy subjects (four men, eight women; 28.6 ± 5.0 years) were recruited for this study. The local ethics committee approved the study and all subjects gave informed written consent. All subjects were healthy without vascular, respiratory, cardiac or neurological disease (self-reported). Each subject was familiarized with the respiratory circuit outside the MR scanner prior to the experimental session. An anaesthetist monitored subjects (peripheral oxygen saturation, respiratory rate and heart rate) during the hypoxic challenges.

### Perfusion measurements

MRI data were collected at 3 T (HDx, General Electric) using an eight-channel receive-only head coil. CBF was estimated using a dual-echo, single-shot, proximal inversion with a control for off-resonance effects – quantitative imaging of perfusion using single subtraction II (PICORE-QUIPSS II) 15 ASL acquisition with gradient-echo spiral readout. Imaging parameters were: TR/TE_1_/TE_2_ = 2200 ms/3 ms/29 ms; TI_1_/TI_2_ = 600 ms/1500 ms; field of view, 22 cm × 22 cm; six g94 slices, 7 mm thick, with a 1-mm gap between slices; matrix, 64 × 64. A 33-min scan (900 repetitions) was performed during the hypoxia protocol (described below). Slices were placed to maximize coverage of the cerebrum with the most inferior slice at the base of the occipital lobe and extending superiorly to include the parietal and temporal lobes.

CBF data were pre-processed using surround subtraction of the ASL tag and control images 16. With the same slice prescription, calibration scans were acquired to provide an estimate of *M*_0_ (fully relaxed blood water magnetisation) 17. *R*_2_* was calculated from the dual-echo data, *R*_2_* = [ln(*S*_1_/*S*_2_)]/(TE_2_ – TE_1_), allowing us to calculate the Δ*R*_2_* time course with respect to the mean *R*_2_* of the initial baseline period of normoxia.

CBF was calculated using a standard single-compartment model 17–19. We incorporated three corrections for alterations of model parameters in hypoxia that are otherwise likely to bias the CBF estimates.

1. The shortening of *T*_2_* in hypoxia. The calculated Δ*R*_2_* time course was used to dynamically correct the ASL signal. This aims to account for the enhanced *T*_2_* decay of the perfusion label, although this effect is expected to be small with the short TE of 3 ms employed 20.
2. The shortening of *T*_1_ of arterial blood. During the initial normoxic baseline period, we assumed arterial blood *T*_1_ = 1.664 s 21. During steady-state hypoxia (final portion of the hypoxic period), we assumed *T*_1_ for arterial blood = 1.611 s, based on the group mean level of blood oxygen desaturation observed in this study and the dependence of *T*_1_ on oxygenation reported in the literature 21. The change in *R*_2_* was used as an index of oxygenation to estimate the arterial blood *T*_1_ at each time point by linear interpolation between 1.611 s and 1.664 s.
3. A reduced arterial arrival time with increasing blood flow. The expected increase in CBF with hypoxia is likely to result in a decrease in the tissue arrival time. This is the time after the labelling pulse at which the labelled spins are assumed to have entered the parenchyma from the brain’s capillaries. A decreased arrival time means that the labelled spins spend less time relaxing at the *T*_1_ of blood and more time relaxing at the *T*_1_ of brain parenchyma, assumed to be 1.165 s 22. Tissue arrival times are expected to vary across the brain 23, but we are unable to estimate this effect, having acquired data only with a single post-label delay. We therefore assume that, in normoxia, the label reaches the tissue at 1500 ms across the whole brain (TI_2_ for the first slice acquired). The estimate of the dependence of the tissue arrival time on global CBF was based on the work of Ho *et al*. 24, in which a 5-ms decrease in arrival time per 1% increase in CBF was observed.

As can be seen in equation 3 of ref. 18, which summarises the signal model, the factor *q*_P_ is dependent on CBF because of the dependence of the arrival time on flow and is dependent on hypoxia through the changing blood *T*_1_. The expression was therefore solved numerically to estimate CBF at each time point using Matlab (The Mathworks Inc., Natick, MA, USA) and a tissue–blood partition coefficient of water (*λ* = 0.9) 18.

A whole-brain *T*_1_-weighted (fast spoiled gradient recalled echo, 1-mm^3^ voxels, TI/TR/TE = 450 ms/7.8 ms/3 ms) image was used for registration. All data were registered to MNI space using FLIRT [FSL, FMRIB – http://www.fmrib.ox.ac.uk/fsl 25] and regional data were extracted using anatomical regions defined by the MNI atlas, available in FSL (regions: frontal lobe, insula, occipital lobe, parietal lobe, putamen, temporal lobe and thalamus).

### Respiratory protocol

Inspired gas concentrations were controlled in the MR scanner using dynamic end-tidal forcing 26. Subjects breathed through a tight-fitting facemask (Quadralite, Intersurgical, Wokingham, Berkshire, UK). Gas partial pressures were measured using rapidly responding gas analysers (Models CD-3A and S-3A; AEI Technologies, Pittsburgh, PA, USA). Dynamic end-tidal forcing was performed using custom software (BreatheDmx, Oxford University, written in LabView, National Instruments, Newbury, Berkshire, UK), which compares the measured end-tidal gases with the desired values and then controls the gas delivery to the subjects on a breath-by-breath basis to meet and maintain the desired end-tidal partial pressure of each gas 26,27. Gas delivery from high-pressure gas cylinders (BOC, Margam, Port Talbot, UK) was controlled with four mass-flow controllers (Model MFC 1559A, MKS Instruments, Wilmington, MA, USA) for medical air, 100% O_2_, 10% O_2_/balance nitrogen and 10% CO_2_/balance air, powered by two two-channel power supplies (Model PR4000, MKS Instruments). An analogue-to-digital data acquisition card (National Instruments Corp., Austin, TX, USA) was used for the acquisition of the gas partial pressures from the gas analysers (recorded at 500 Hz) and for the digitization of the flow direction from an MR-compatible flow transducer (VMM-400, Interface Associates, Laguna Niguel, CA, USA). Gases flowed continuously, passing through a mixing chamber placed close to the volunteer to ensure adequate and fast mixing. The inspirate was drawn from this continually flowing gas stream. The sampling port for the establishment of CO_2_ and O_2_ levels was positioned on the port of the facemask. This design ensures minimal delay in the delivery of an updated gas mixture and minimal mixing of expired and inspired gases within the constraints of the end-tidal forcing system 26.

**Figure 1:**
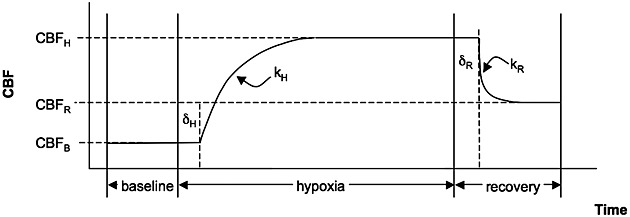
Schematic diagram of modelling parameters. This model was used for both the cerebral blood flow (CBF) and *R*_2_* time series but, for descriptive purposes, we refer here to CBF only. CBF_B_ is the CBF calculated during the baseline period, and CBF_H_ and CBF_R_ are the equilibrium CBF values obtained during hypoxia and recovery, respectively. The rate constants during the transition to hypoxia and back to normoxia during the recovery are denoted by *k*_H_ and *k*_R_, respectively, and these transitions occur at delays of *δ*_H_ and *δ*_R_ after the gas mixtures are switched to the hypoxic challenge or back to normoxia, respectively.

Resting partial pressures of end-tidal CO_2_ and O_2_ (*P*_ET_CO_2_ and *P*_ET_O_2_, respectively) were established after the subject had acclimatized to the experimental set-up but prior to the ASL acquisition. The hypoxic protocol, based on that of Poulin *et al*. 8, lasted 33 min and consisted of a 5-min baseline (normoxia), 20 min of hypoxia (target *P*_ET_O_2_ = 50 mmHg) and 8 min of recovery (normoxia) (see Fig. 1). The end-tidal forcing system aimed to maintain *P*_ET_CO_2_ at the subject’s resting value throughout.

### Temporal response model

In order to quantify the magnitude and temporal dynamics of the CBF and *R*_2_* responses, a temporal model similar to that of Poulin *et al*. 8 was applied. We refer to CBF in the description that follows; however, the same model was also applied to the *R*_2_* time course. The hypoxic model parameters included: CBF at baseline normoxia (CBF_B_); an exponential increase defined by the rate constant *k*_H_ with the CBF increase beginning at a delay *δ*_H_ after the onset of hypoxia, leading to a plateau CBF (CBF_H_) during hypoxia; and, similarly, recovery during normoxia beginning at a delay *δ*_R_ and described by an exponential rate constant (*k*_R_) reaching a recovery CBF (CBF_R_). (See Fig. 1 for a schematic description of these parameter definitions.) All parameters were determined using nonlinear fitting, and the 95% confidence intervals (95% CIs) of all the parameter fits were estimated (Matlab) as a reflection of the influence of noise in the data on the fitted parameters. The minimum values for the delay times *δ*_H_ and *δ*_R_ were constrained to 20 s for CBF and to 0 s for *R*_2_*. There was an inherent finite transition period of 20 s between the inspired oxygen levels imposed by the function of the end-tidal forcing system. We allowed our model to describe *R*_2_* changes without delay because *R*_2_* would be expected to change as soon as the blood oxygenation begins to transition. However, CBF is expected to rise on the transition to hypoxia, for example, only after a substantial drop in oxygenation has occurred, and the end-tidal forcing system would not bring this about until after its 20-s inherent transition period.

Before fitting the time-series data to the model, in order to improve the signal-to-noise ratio (SNR) in the CBF measurement, registered individual subject data were averaged to produce a group-averaged time course. The hypoxia time-series model was applied to whole-brain grey matter and on a regional basis using the MNI structural atlas to define the frontal lobe, insula, occipital lobe, parietal lobe, putamen, temporal lobe and thalamus. As a supplementary investigation, the model was also used to characterise the hypoxic response in the whole-brain grey matter of individual subjects.

### Reactivity

Hypoxic reactivity was calculated on an individual subject basis as the percentage change in CBF or the absolute change in *R*_2_* from the last 5 min of the hypoxic period compared with the 5 min of baseline normoxia normalized by the absolute change in *P*_ET_O_2_.

### Statistical analysis

Differences in physiological data (*S*_p_o_2_, *P*_ET_O_2_, *P*_ET_CO_2_, heart rate and ventilation rate) between normoxia and hypoxia were tested using paired *t*-tests of the individual average data during normoxic and the last 5 min of the hypoxic condition. Two-way analysis of variance (ANOVA) was used to assess the effect of region and subject on the regional reactivity for both the CBF and *R*_2_* data.

## RESULTS

All subjects tolerated the hypoxic challenges well. The hypoxic challenge was stopped early in one subject because of a technical difficulty and, as a result, the data from the final ∼10 min of this session were excluded from the analysis.

**Figure 2:**
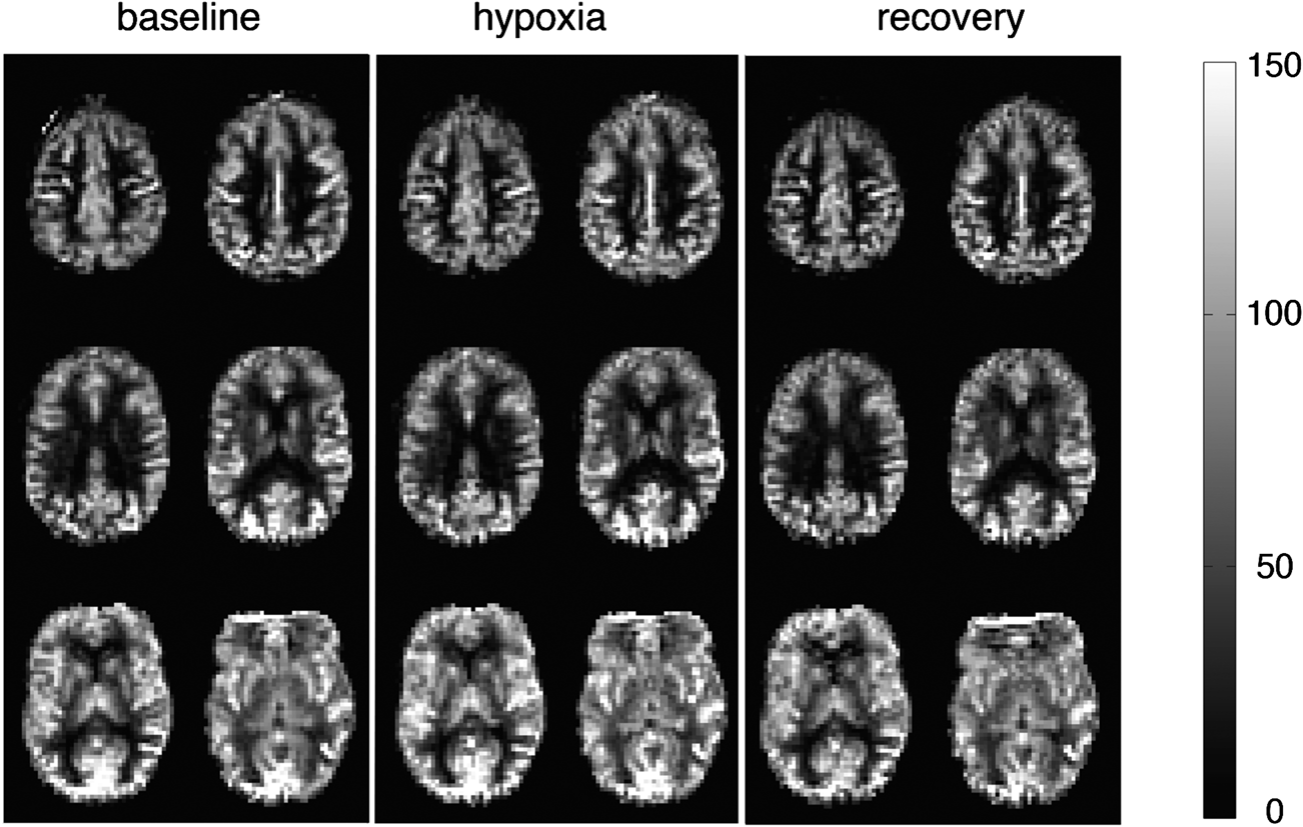
Sample cerebral blood flow (CBF) maps from one individual during normoxic baseline (a), hypoxic hypoxia (b) and normoxic recovery (c). Each map is derived from 5 min of data and has been registered to MNI space. CBF is displayed in mL/100 g tissue/min.

**Figure 3:**
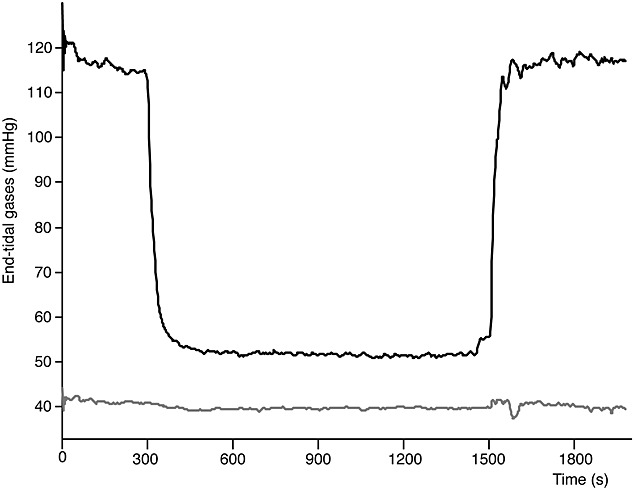
Group-averaged end-tidal O_2_ (black) and CO_2_ (grey).

During the hypoxic challenge, according to pulse oximetry, subjects desaturated to an average *S*_p_o_2_ of 83%, with the subject-averaged data reaching this level after 810 s. Group-averaged total oxygen delivery at baseline was 15.1 mL/100 g/min and, in hypoxia, was 14.7 mL/100 g/min, assuming that the dissolved oxygen is negligible 13 and that the concentration of haemoglobin (assumed to be 15 g/dL) does not change during the challenge. A summary of the physiological data is shown in Table 1. Sample CBF maps are shown in Fig. 2, and group-averaged end-tidal O_2_ and CO_2_ are shown in Fig. 3. Although the inclusion of the dynamic changes in *T*_1_, *R*_2_* and tissue arrival times is expected to reduce bias in the estimates for CBF in hypoxia, these refinements have the capacity to add noise in the calculated CBF time series, for example, when noise is already present in the *R*_2_* estimate as a result of head motion. As a result of nonconvergence of the numerical estimates of CBF, time-course data from three regions from one subject and one from another were excluded from the regional CBF estimates.

**Table 1:** Summary of physiological measurements between baseline and hypoxic conditions (shown as mean ± standard deviation over subjects). The baseline average is across the entire 5-min baseline normoxia period and the hypoxic average is the average across the last 5 min of the hypoxic period

	Baseline	Hypoxia	*p*
*S*_p_o_2_ (%)	98.5^a^	83.4 ± 6.8	
Respiration rate (breaths/min)	13.2 ± 4.5	14.3 ± 3.7	0.05
Heart rate (beats/min)	66.6 ± 7.9	74.4 ± 8.7	<0.001
*P*_ET_co_2_ (mmHg)	41.2 ± 2.8	39.8 ± 2.5	<0.001
*P*_ET_o_2_ (mmHg)	116.6 ± 4.4	52.0 ± 3.8	<0.001

*P*_ET_co_2_, partial pressure of end-tidal carbon dioxide; *P*_ET_o_2_, partial pressure of end-tidal oxygen; *S*_p_o_2_, blood oxygen saturation.

*p* values are derived from a paired *t*-test comparing baseline normoxia with hypoxic hypoxia.

a Assumed from standard values.

**Figure 4:**
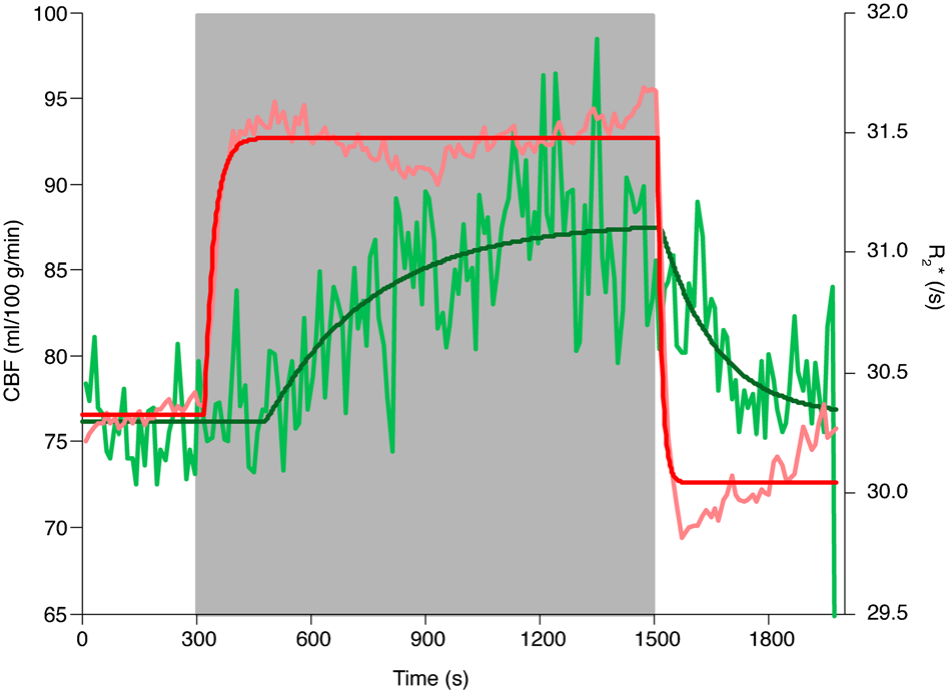
Cerebral blood flow (CBF) and *R*_2_* across whole-brain grey matter. Quantified CBF and the CBF modelled response are shown in light green and dark green, respectively. R_2_* over time and the modelled response are shown in pink and red, respectively. The time-series data for CBF and R_2_* were averaged into 11-s time bins for display purposes.

According to the model fitting of the group-averaged data, across grey matter, CBF increased from 76.1 mL/100 g/min (95% CI: 75.5, 76.7) to 87.8 mL/100 g/min (95% CI: 86.7, 89.6), with a rate constant of 0.0035 s^–1^ (95% CI: 0.0019, 0.0046) and a delay of 185 s (95% CI: 132, 230). This corresponds to a 15.4% increase in CBF. The parameters from the fitting (and the 95% CIs on these fits showing the reliability or noise in the parameter fit) across the regions are given in Table 2. The CBF and *R*_2_* time-course and model fits for all grey matter are shown in Fig. 4. The *R*_2_* response was more rapid than the CBF response, with a rate constant of 0.0392 s^–1^ and a delay of 24 s calculated for the transition to hypoxia (Table 3, Fig. 4).

**Table 2:** Model parameters (confidence intervals of fitting) for cerebral blood flow (CBF) response to hypoxia

	CBF_B_ (mL/100 g/min)	CBF_H_ (mL/100 g/min)	CBF_R_ (mL/100 g/min)	*k*_H_ (s^–1^)	*k*_R_(s^–1^)	*δ*_H_ (s)	*δ*_R_ (s)
All grey matter	76.1	87.8	76.4	0.0035	0.0069	185	20^*^
	(75.5, 76.7)	(86.7, 89.6)	(73.8, 78.7)	(0.0019, 0.0046)	(0.002, 0.0103)	(132, 230)	(–38,38)
Frontal lobe	56.9	68.2	57.0	0.0032	0.005	250	20[Table-fn tf2-1]
	(56.4, 57.4)	(66.8, 69.7)	(53.6, 60.5)	(0.0018, 0.0046)	0.0008, 0.0091)	(202, 298)	(–26, 65)
Insula	71.1	83.4	73.9	0.0034	0.0132	83	121
	(70.0, 72.2)	(82.0, 84.5)	(71.6, 76.2)	(0.0017, 0.005)	(-0.0019, 0.0284)	(12, 153)	(80, 163)
Occipital lobe[Table-fn tf2-2]	89.8	101.8	89.5	0.0035	0.0099	46	20
	(88.9, 90.7)	(100.6, 102.9)	(87.9, 91.1)	(0.0021, 0.005)	(0.0038, 0.016)	(–8, 100)	(–10, 50)
Parietal lobe[Table-fn tf2-2]	66.7	76.3	65.9	0.0042	0.0062	159	88
	(66.1, 67.2)	(75.6, 77.0)	(62.6, 69.2)	(0.0027, 0.0058)	(0.0008, 0.0116)	(115, 203)	(49, 128)
Putamen[Table-fn tf2-2]	62.8	72.5	60.9	0.003	0.0052	83	30
	(62.1, 63.4)	(71.4, 73.6)	(57.6, 64.2)	(0.0018, 0.0043)	(0.0012, 0.0093)	(26, 140)	(–10, 70)
Temporal lobe	77.4	87.1	77.1	0.0029	0.0062	85	20
	(76.5, 78.2)	(85.5, 88.8)	(73.9, 80.3)	(0.0012, 0.0045)	(0.000, 0.0124)	(7, 163)	(–34, 74)
Thalamus[Table-fn tf2-2]	62.4	71.8	59.3	0.0044	0.0098	67	20
	(61.8, 63.1)	(71.0, 72.5)	(58.0, 60.6)	(0.0027, 0.0062)	(0.0051, 0.0144)	(20, 115)	(–3, 43)

CBF_B_, baseline CBF; CBF_H_, equilibrium CBF during hypoxia; CBF_R_, recovery CBF; *k*_H_, rate constant in the transition to hypoxia; *k*_R_, rate constant in the return to normoxia; *δ*_H_, delay in hypoxia response; *δ*_R_, delay in response on return to normoxia (refer to Fig. 1).

Delay value fitted at the lower boundary.

Regional data based on 11 subjects.

**Table 3:** Model parameters (confidence intervals of fitting) for *R*_2_^*^ response to hypoxia

	*R*_2_^*^_B_ (s^–1^)	*R*_2_^*^_H_ (s^–1^)	*R*_2_^*^_R_ (s^–1^)	*k*_H_ (s^–1^)	*k*_R_ (s^–1^)	*δ*_H_ (s)	*δ*_R_ (s)
All grey matter	30.33	31.48	30.04	0.0392	0.0921	24	12
	(30.31, 30.34)	(31.47, 31.49)	(30.03, 30.06)	(0.0333, 0.045)	(0.0744, 0.1098)	(21,26)	(10,13)
Frontal lobe	34.45	35.41	33.74	0.0577	0.1041	22	10
	(34.42, 34.48)	(35.40, 35.43)	(33.7173, 33.7703)	(0.0345, 0.0809)	(0.0701, 0.1382)	(17,27)	(8,12)
Insula	19.01	19.85	18.44	0.0239	0.0864	22	9
	(19.00, 19.02)	(19.84, 19.85)	(18.44, 18.45)	(0.0221, 0.0256)	(0.0796, 0.0932)	(20,24)	(8,9)
Occipital lobe	30.14	31.68	30.09	0.0091	0.0648	0	12
	(30.12, 30.16)	(31.67, 31.69)	(30.07, 30.11)	(0.0085, 0.0096)	(0.0547, 0.0749)	(–5, 5)	(10,14)
Parietal lobe	23.47	24.86	23.12	0.0135	0.0847	9	11
	(23.46, 23.48)	(24.85, 24.86)	(23.11, 23.13)	(0.0127, 0.0143)	(0.0755, 0.0939)	(6,12)	(10,11)
Putamen	31.84	32.58	31.80	0.1363	0.1449	32	20
	(31.81, 31.87)	(32.56, 32.59)	(31.77, 31.82)	(0.0377, 0.235)	(0.0182, 0.2717)	(28, 35)	(16,25)
Temporal lobe	33.77	35.33	33.45	0.0241	0.1114	13	10
	(33.73, 33.79)	(35.32, 35.34)	(33.42, 33.47)	(0.0208, 0.0274)	(0.0837, 0.1392)	(9,17)	(8,12)
Thalamus	21.36	22.97	20.95	0.0217	0.082	18	11
	(31.35, 21.37)	(22.96, 22,98)	(20.94, 20.96)	(0.0204, 0.0229)	(0.075, 0.0891)	(16,20)	(10,11)

*R*_2_^*^_B_, baseline *R*_2_^*^; *R*_2_^*^_H_, equilibrium *R*_2_^*^ during hypoxia; *R*_2_^*^_R_, recovery *R*_2_^*^; *k*_H_, rate constant in the transition to hypoxia; *k*_R_, rate constant in the return to normoxia; *δ*_H_, delay in hypoxia response; *δ*_R_, delay in response on return to normoxia (refer to Fig. 1).

Reactivity, summarized in Table 4, was calculated from the average CBF from the last 5 min of hypoxia compared with baseline normoxia. The two-way ANOVA examining the effects of subject and region on CBF did not show any differences across regions, but did detect a subject effect (*p* < 0.0001). For the *R*_2_* data, both subject (*p* < 0.0001) and region (*p* = 0.02) were significant factors explaining the variance in regional reactivity.

**Table 4:** Regional hypoxic reactivity [negative values indicate increasing cerebral blood flow (CBF) and *R*_2_^*^ with decreasing oxygen levels]

	Reactivity (% ΔCBF/Δ mmHg)	*R*_2_^*^ reactivity (Δ*R*_2_^*^ s^–1^/Δ mmHg)
All grey matter	–0.2548 ± 0.1275	–0.0211 ± 0.0157
Frontal lobe	–0.3187 ± 0.1424	–0.0151 ± 0.0239
Insula	–0.2880 ± 0.1931	–0.0135 ± 0.0081
Occipital lobe	–0.1650 ± 0.1849	–0.0258 ± 0.0078
Parietal lobe	–0.2345 ± 0.0947	–0.0232 ± 0.0103
Putamen	–0.2253 ± 0.1085	–0.0152 ± 0.0269
Temporal lobe	–0.1919 ± 0.1103	–0.0272 ± 0.0198
Thalamus	–0.1997 ± 0.1441	–0.0265 ± 0.0095

## DISCUSSION

We have described the dynamics of the CBF response to acute hypoxic hypoxia in a cohort of healthy, young adults. Although the CBF amplitude response to hypoxia has been investigated previously using ASL 12,13, to our knowledge, this is the first time the temporal dynamics of the global and regional CBF response have been quantified, and compared with a simultaneously acquired marker of cerebral blood oxygenation (*R*_2_*).

Across grey matter, the time-course model indicated that CBF increased from 76.1 mL/100 g/min to 87.8 mL/100 g/min (Table 2), showing a 15.4% increase in CBF during the hypoxic challenge, which is consistent with the observed increases in CBF during similar challenges 11–13. An exponential model using a normoxic baseline and a single hypoxic condition has been applied previously to describe the dynamics of the arterial blood flow response to a similar hypoxic challenge (target *P*_ET_O_2_ = 50 mmHg) using TCD to measure blood flow in the MCA 8. The study of Poulin *et al*. 8 used two rate constants (one for the transition to hypoxia and another for the return to normoxia) and a common delay term for the blood velocity response for both the transition from normoxia to hypoxia and the transition from hypoxia to normoxia. Here, we extend this approach to include separate delay terms for the transition to hypoxia and the return to normoxia. This additional model flexibility removes any assumptions of symmetry between the blood flow response to hypoxia and return to normoxia after the hypoxic challenge. In the present study, the rate constant for the CBF response to hypoxia across grey matter was 0.0035 s^–1^ and the delay for this response was 185 s (Table 2). The rate constant and delay for the return to normoxia were 0.0069 s^–1^ and 20 s (not significantly different from the 0-s delay as indicated by the 95% CIs), respectively, both faster and shorter than the transition to hypoxia, consistent with the observations in other studies 8,13. After a 20-min hypoxic challenge, there is evidence that blood flow in the MCA remains mildly increased for at least 5 min 28, which may indicate that a steady-state condition is not reached within our 8-min observation period during recovery.

Previously reported temporal parameters of flow in the MCA show a high degree of variability 8. We have presented a group-averaged analysis in order to increase the SNR to assist model fitting to the data; however, uncertainty associated with the fitted parameters remains. For example, natural individual heterogeneity may be a dominant source of rate constant variability. The analysis performed on the group-averaged data was also applied to individual data (Table S1). Although there appear to be some outliers and some failures to estimate CBF, these data illustrate potential inter-individual differences in the CBF response to hypoxia. The rate constant for the return to normoxia after the hypoxic challenge is more variable than that of the response to hypoxia, both within and across regions (see CIs in Table 2).

TCD studies estimating the temporal characteristics of the blood flow response to hypoxia have found higher rate constants and shorter delays (i.e. a faster transition) than those observed here 8,28 and qualitatively observed in a previous ASL study of hypoxia [see figs 3–5 of ref. 13]. Poulin *et al*. 8 reported an average time constant of 79.6 s, equivalent to a rate constant of 0.0126 s^–1^; however, on removing data from two volunteers with measured time constants of less than 1 s, two orders of magnitude less than the mean, the equivalent rate constant is ∼0.008 s^–1^, more similar to the values observed here. Nevertheless, a difference between the dynamics estimated by Poulin *et al*. 8 and the present experiment remains. As a result of the experimental constraints of end-tidal forcing in the MR environment, we chose a minimum 20-s transition period for changes in target end-tidal values, which may affect the rate of the CBF response. This could suggest a nonlinear relationship between the speed of hypoxia onset and the rate of the CBF response. Perhaps a faster hypoxia onset, as used by Poulin *et al*. 8, stimulates a faster blood flow response. Alternatively, differences in the temporal SNR may result in longer delays being estimated at lower SNR. TCD measures of CBF based on bulk arterial blood velocity may differ from CBF based on the ASL signal that arises from water that has entered the brain tissue. Furthermore, it is common that, in TCD measurements, the MCA is assumed not to change diameter; thus changes in blood velocity are assumed to be proportional to changes in blood flow. However, a recent study using multiple imaging modalities (TCD and phase-contrast MRI) has shown that the MCA changes diameter after an acute (180 min) hypoxic challenge and chronic hypoxic exposure 3, which could affect the TCD estimates of dynamic changes in flow.

Four reflexes are implicated in the blood flow response to hypoxia: (i) the hypoxic ventilatory response; (ii) the hypercapnic ventilatory response; (iii) hypoxic vasodilation; and (iv) hypocapnic vasoconstriction. These responses interact, increasing the system complexity 1. Although there are multiple mechanisms of vasodilation, adenosine is thought to be a main mediator of hypoxic-induced vasodilation 1, and its concentration parallels the increase in CBF during hypoxic exposure 29. Additional mediators include potassium and calcium ions and nitric oxide 1. The hypoxic ventilatory response is centrally mediated and modulated by CO_2_, which is detected by both central and peripheral chemoreceptors 1. These interactions may be a cause of the delay of the CBF response to the hypoxic stimulus. Cerebral autoregulation has been shown to remain intact in acute hypoxia 30 and, more recently 31, inconsistencies in reports have been attributed to CO_2_ variation and have confirmed that cerebral autoregulation is also maintained during hypocapnic hypoxia.

We used the overall *R*_2_* as an indicator of changes in blood oxygenation, as *R*_2_* is dependent on the concentration of deoxyhaemoglobin in arterial and venous blood. This was then used as an index of changes in *T*_1_ of arterial blood. As expected, *R*_2_* responded rapidly to changes in the inspired oxygen levels, confirming that end-tidal forcing is effective in rapidly inducing changes in arterial and venous oxygenation. The time-course data of the *R*_2_* and CBF changes indicate consistency between these two metrics during the hypoxic challenge, although there are differences in the speed of response (Fig. 4). With the onset of hypoxic hypoxia, *R*_2_* increases rapidly as a result of the decrease in blood oxygenation, both arterial and venous. Approximately 3 min (180 s, Table 2) after the onset of hypoxia (time ∼ 480 s in Fig. 4), CBF begins to increase and, thereafter, *R*_2_* decreases slightly (500–900 s, Fig. 4), which is consistent with the increasing CBF resulting in increased cerebral venous oxygenation. However, an increase in cerebral blood volume associated with the increase in CBF would tend to oppose this effect, perhaps explaining the subsequent increase in *R*_2_* between 900 and 1500 s (Fig. 4). At the end of the hypoxic challenge, *R*_2_* decreases rapidly, as arterial and venous oxygenation increase towards normal (*P*_ET_O_2_ ∼ 110 mmHg). Again, the CBF response is slower than the *R*_2_* response. After approximately 150 s, *R*_2_* begins to increase slightly as the venous deoxyhaemoglobin concentration rises, potentially as a result of the CBF decrease.

The use of dynamic end-tidal forcing enables the examination of the effects of hypoxia with the advantage of being able to perform well-controlled respiratory challenges. Despite the end-tidal forcing system’s attempted control over *P*_ET_CO_2_, there was an average 1.3 mmHg decrease in *P*_ET_CO_2_ during the hypoxic challenge (Table 1). Hypocapnia decreases blood flow by 2–3%/mmHg 1; thus an underestimation of the hypoxic CBF response of up to approximately 4% may be present.

The inclusion of the effects of changing *T*_2_*, arterial blood *T*_1_ and tissue arrival time had a substantial effect on the estimate of CBF change with hypoxia. Without the inclusion of these factors, CBF would be estimated to increase by approximately 9%, in comparison with the 15% increase reported here with these factors included. The effect of hypoxia on shortening arterial blood *T*_1_ and the assumed effect of the shortened arrival time had the most substantial effect on the estimate of the CBF increase, whereas the correction for shortened *T*_2_* played a minor role. *T*_1_ and the arrival time impact directly on the calculation of the longitudinal magnetization. Without these corrections, the flow increase is underestimated, as the overall *T*_1_ decay of the label at the point of signal acquisition is underestimated. The effect of *T*_2_* was particularly small (**∼**3.7% change in *T*_2_* between normoxia and hypoxia) because of the short TE (3 ms) of our spiral acquisition, TE being defined as the point at which the centre of *k* space is traversed. There remains the possibility of un-modelled *T*_2_* weighting of the CBF signal as the acquisition window naturally extends beyond 3 ms. However, our focus on regional and whole-brain analyses (low spatial frequency) means that our results will be influenced most by data collected in the centre of *k* space rather than the high-frequency data collected later in the acquisition. Furthermore, the majority of the power of the signal in *k* space is acquired within 6 ms of the excitation. If we assume an effective time of signal acquisition of 6 ms, the subsequent error in the reported CBF estimate would be less than 1%.

Using nuclear medicine imaging methods, others 2,10,11 have detected regional differences in the magnitude of the CBF response to hypoxia. However, the regions that show increased blood flow during hypoxia differed between these studies. Binks *et al*. 11 observed different responses regionally, and attributed these differences to differing phylogenetic ages of the regions, specifically that ‘older’ brain regions have an increased CBF response compared with ‘newer’ regions. Pagani *et al*. 10 reported a greater hypoxic CBF response in the sensory motor cortex, anterior cingulate cortex, prefrontal cortex, basal ganglia and right temporal lobe. Some of these regions are components of attentional networks, and Pagani *et al*. 10 suggested that, because attention is a basic, energy-consuming process, these networks are protected in acute hypoxia. The resolution of the discrepancies in differences in the CBF response is further confounded by the observation that arteries feeding the brain have different sensitivities to hypoxia 7. Nevertheless, regional differences in the CBF response to hypoxia have interesting implications in terms of cerebrovascular physiology and vulnerability to hypoxic damage. Therefore, in addition to examining the hypoxic response across the entire brain, we also analysed the hypoxic response regionally. As a result of limited brain coverage, and with the need in mind to perform spatial signal averaging to maintain signal-to-noise for model fitting, we restricted our regional analysis to seven broad regions. We did not detect regional differences in CBF reactivity. However, regional differences in *R*_2_* reactivity were significant. Subject was a significant factor for both CBF and *R*_2_* reactivity, but the inter-subject differences did not appear to be explained by the small differences in the level of *P*_ET_O_2_ reached (data not shown). Therefore, it appears that individuals may have notably different levels of overall hypoxic reactivity. This inter-subject heterogeneity reduces our ability to detect inter-regional differences in reactivity to hypoxia across the group, but may also be important in determining the effects on an individual of a hypoxic event.

## CONCLUSIONS

In this study, we quantified the dynamic CBF response to hypoxia during an acute (20 min) hypoxic challenge. We characterised both the magnitude and temporal characteristics of the CBF response across all grey matter and on a regional basis, and found that, across grey matter, CBF increased by approximately 15% at mean *P*_ET_O_2_ = 52 mmHg. In addition, we used *R*_2_* as a simultaneous marker of local blood oxygenation. In this healthy population, the CBF response to hypoxia was relatively slow, with a delay to onset of approximately 3 min and a time constant of approximately 4.5 min. In contrast, the CBF response on the return to normoxia from hypoxia was more rapid. The blood oxygenation response was much more rapid and was contemporaneous with the onset of the hypoxic challenge. Broad regional differences in the CBF response to hypoxia were not detected. However, individual heterogeneity may have a substantial role in determining the CBF response to hypoxia.

## Abbreviations

ANOVA: analysis of variance
ASL: arterial spin labelling
CBF: cerebral blood flow
CI: confidence interval
MCA: middle cerebral artery
P_a_o_2_: arterial partial pressure of oxygen
P_ET_co_2_: partial pressure of end-tidal carbon dioxide
P_ET_o_2_: partial pressure of end-tidal oxygen
SNR: signal-to-noise ratio
_p_o_2_: blood oxygen saturation
TCD: transcranial Doppler
