# Supplementary material for: Cerebral blood flow response to acute hypoxic hypoxia
Source: NMR Biomed. 2013 Oct 7;26(12):1844–52. doi: 10.1002/nbm.3026 (PMC4114548; doi:10.1002/nbm.3026)
Supplement: Supplementary file 2 — Supporting information may be found in the online version of this article. [file nbm-26-1844-sd1.docx]

**Supplementary Table**. Model parameters for individual subjects for grey matter CBF. Using the same model applied to the group average data, individual response parameters were quantified. Unfortunately the reduced SNR of this approach limits the interpretation of this data; however, it is nonetheless important to note the individual heterogeneity of the hypoxic response parameters.

|  | CBF_B_  (mL/100 g /min) | CBF_H_  (mL/100 g /min) | CBF_R_  (mL/100 g /min) | k_H_  (/s) | k_R_ (/s) | δ_H_  (s) | δ_R_ (s) |
| --- | --- | --- | --- | --- | --- | --- | --- |
| S01 | 76.3 | 86.4 | 43.7 | 0.0039 | 0.0009 | 228 | 46 |
| S03 | 81.9 | 91.5 | 80.2 | 0.0148 | 0.0497 | 228 | 20 |
| S04 | 85.4 | 94.5 | 82.3 | 0.0077 | 0.0092 | 300 | 20 |
| S05 | 76.1 | 89.8 | 82.5 | 0.4545* | 0.4499 | 32 | 182 |
| S06 | 56.9 | 74.3 | 65.6 | 0.0008 | 0.3310 | 91 | 135 |
| S07 | 75.6 | 85.6 | 80.3 | 0.0036 | 0.4180 | 300 | 253 |
| S08 | 56.2 | 95.3 | 56.9 | 0.0008 | 0.0866 | 32 | 20* |
| S09 | 62.6 | 132.0 | 54.6 | 0.0004 | 0.0046 | 259 | 240 |
| S10 | 61.5 | 72.5 | 40* | 0.0064 | 0.0012 | 20* | 20* |
| S11 | 76.7 | 88.5 | 70.8 | 0.2387 | 0.0119 | 298 | 62 |
| S12 | 70.9 | 91.0 | 65.7 | 0.07316 | 0.1363 | 179 | 100 |

Note subject 02 was not included in this analysis as the hypoxic challenge was stopped early, parameters that were estimated at the boundaries included in the non-linear signal fitting routine are marked with an *
